# Supplementary material for: Male-Specific Protein Disulphide Isomerase Function is Essential for Plasmodium Transmission and a Vulnerable Target for Intervention
Source: Sci Rep. 2019 Dec 4;9:18300. doi: 10.1038/s41598-019-54613-0 (PMC6892906; doi:10.1038/s41598-019-54613-0)
Supplement: Supplementary file 1 — Dataset 1 [file 41598_2019_54613_MOESM1_ESM.pdf]

Male-Specific Protein Disulphide Isomerase Function is Essential for *Plasmodium* Transmission and a Vulnerable Target for Intervention

Fiona Angrisano[1,] Katarzyna A. Sala[2], Sofia Tapanelli[2], George K. Christophides[2], Andrew M. Blagborough[1, 2, 3*]

S1.

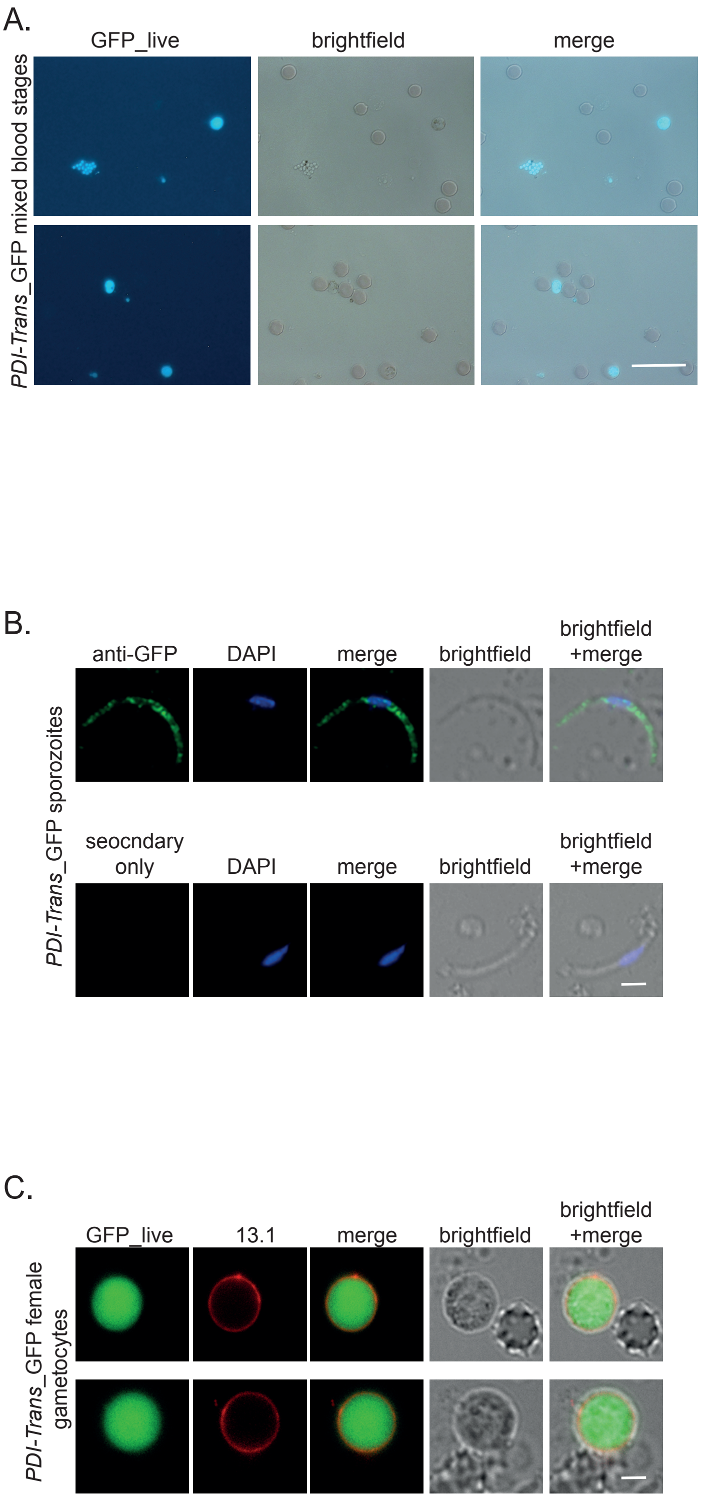
A.
GFP_live
brightfield
merge
PDI-Trans_GFP mixed blood stages
B.
anti-GFP
DAPI
merge
brightfield
brightfield +merge
seocndary only
DAPI
merge
brightfield
brightfield +merge
PDI-Trans_GFP sporozoites
C.
GFP_live
13.1
merge
brightfield
brightfield +merge
PDI-Trans_GFP female gametocytes

S2.

A.

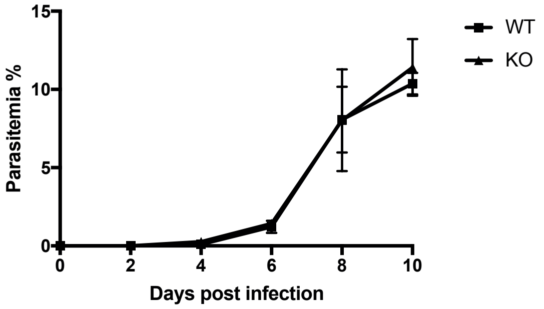

WT
KO
Parasitemia %
15
10
5
0
0
2
4
6
8
10
Days post infection

B.

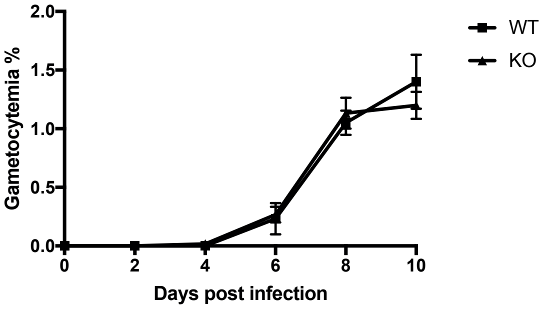

WT
KO
Gametocytemia %
2.0
1.5
1.0
0.5
0.0
0
2
4
6
8
10
Days post infection

S3.

A.

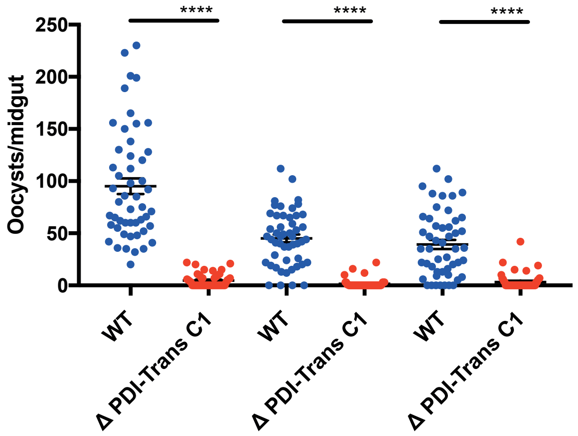

****
****
****
250
200
150
100
50
0
Oocysts/midgut
WT
Δ PDI-Trans C1
WT
Δ PDI-Trans C1
WT
Δ PDI-Trans C1

B.

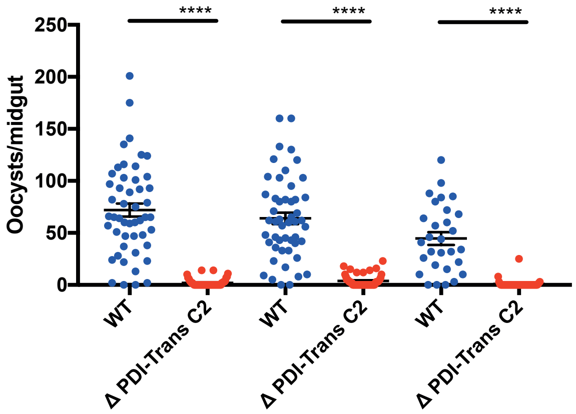

****
****
****
250
200
150
100
50
0
Oocysts/midgut
WT
Δ PDI-Trans C2
WT
Δ PDI-Trans C2
WT
Δ PDI-Trans C2

S4.

A.

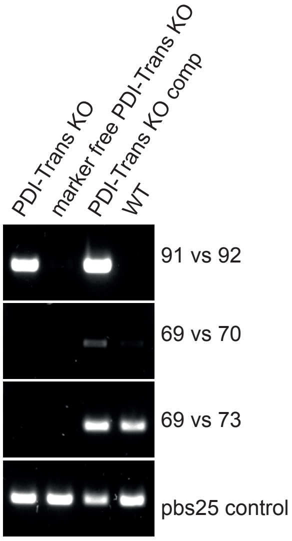
PDI-Trans KO
marker free PDI-Trans KO
PDI-Trans KO comp
WT
91 vs 92
69 vs 70
69 vs 73
pbs25 control

B.

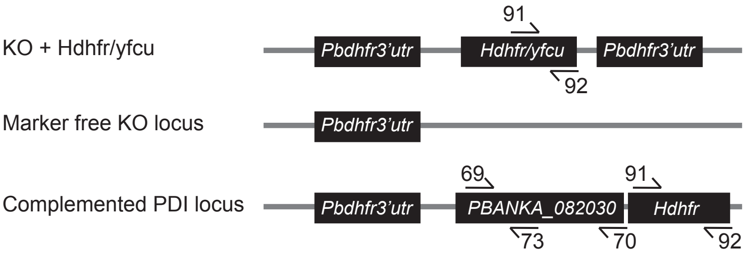
KO + Hdhfr/yfcu
Pbdhfr3'utr
91
Hdhfr/yfcu
92
Pbdhfr3'utr
Marker free KO locus
Pbdhfr3'utr
Complemented PDI locus
Pbdhfr3'utr
69
PBANKA_082030
73
70
91
Hdhfr
92

Supplemental Figure S1.

A). Live GFP fluorescence of mixed blood stage *P. berghei PDI-Trans-GFP* parasites. Scale bar = 15 μm. ***B).*** IFA of fixed, non-permeablised *PDI-Trans-GFP* salivary gland sporozoites probed with either anti-GFP (top) or secondary only (bottom). Each panel shows an overlay of GFP fluorescence (green) and DNA labelled with DAPI (blue). Scale bar = 5 μm. ***C).*** Live GFP fluorescence of *P. berghei* female gametocytes. Parasites were incubated with anti-Pbs28 Cy3 conjugated surface antibodies 13.1 prior to imaging as a co-stain for activated female gametes. Scale bar = 5 μm.

Supplemental Figure S2.

A). Asexual growth and ***B).*** gametocyte production of WT and *ΔPDI-Trans* parasites strains. Three independent experiments are plotted.

Supplemental Figure S3.

Mice infected with ***A).*** *ΔPDI-Trans* clone 1 (C1) or ***B).*** *ΔPDI-Trans* clone 2 (C2) *P. berghei* parasites and DFA performed to determine transmission blockade. Individual data points represent the number of oocysts found in individual mosquitoes 12 days post feeding. Horizontal bars indicate mean intensity of infection, while error bars indicate SEM within individual samples. Asterisks indicate P value < 0.05 Mann-Whitney U test.

Supplemental Figure S4

A). Genotyping data for *ΔPDI-Trans, ΔPDI-Trans* marker-free and *ΔPDI-Trans* Comp lines. ***B).*** Schematic of for *ΔPDI-Trans, ΔPDI-Trans* marker-free and *ΔPDI-Trans* Comp lines and primer pairs used for genotyping.
